# Supplementary material for: Role of Gln79 in Feedback Inhibition of the Yeast γ-Glutamyl Kinase by Proline
Source: Microorganisms. 2021 Sep 7;9(9):1902. doi: 10.3390/microorganisms9091902 (PMC8472793; doi:10.3390/microorganisms9091902)
Supplement: Supplementary file 1 [file microorganisms-09-01902-s001.zip › microorganisms-1361528-supplementary.pdf]

Supplementary Materials

Role of Gln79 in feedback inhibition of the yeast γ-glutamyl kinase by proline

Akira Nishimura [1,†], Yurie Takasaki [1,†], Shota Isogai [1,†], Yoichi Toyokawa [1], Ryoya Tanahashi [1] and Hiroshi Takagi [1,*]

[1]Division of Biological Science, Graduate School of Science and Technology, Nara Institute of Science and Technology, Nara, Japan; nishimura@bs.naist.jp (A. N.), takasaki.yurie.tw6@bs.naist.jp (Y. T.), s-isogai@bs.naist.jp (S. I.), y-toyokawa@bs.naist.jp (Y.T.), tanahashi.ryoya.ti3@bs.naist.jp (R. T.), hiro@bs.naist.jp (H. T.)

[†]The authors contributed equally to this work.

[*]Correspondence: hiro@bs.naist.jp; Tel.: +81-743-72-5420

This file includes:

Table S1. Primers used in this study.

Figure S1. Biosynthesis and degradation of proline in *S. cerevisiae*.

Figure S2. Sequence alignment around Gln79 in Pro1 among various microorganisms.

Figure S3. Purity of the recombinant Pro1 (WT, Q79H, and I150T) proteins.

Figure S4. Purity of the recombinant Pro1 (WT, Q79A, Q79E, Q79K, Q79N, Q79R, and Q79W) proteins.

Figure S5. Uncropped images for Western blot gels of Figure 5 (a).

Figure S6. Glutamate content in yeast cells expressing the Gln79 variants of Pro1.

Figure S7. Sequence alignment around Asp143 and Ser146 in Pro1 among various microorganisms.

Figure S8. Purity of the recombinant Pro1 (WT and D143A) proteins.

Table S1. Primers used in this study.

Name	Sequence (5'-3')	Description
Pro1 Q79A Fw	AGCAGAAGTTGCGGCCATCGCAG	For construction of Pro1 Q79A mutant
Pro1 Q79A Rv	CTGCGATGGCCGCAACTTCTGCT	
Pro1 Q79E Fw	AGCAGAAGTTGAGGCCATCGCAG	For construction of Pro1 Q79E mutant
Pro1 Q79E Rv	CTGCGATGGCCTCAACTTCTGCT	
Pro1 Q79H Fw	AGCAGAAGTTCACGCCATCGCAG	For construction of Pro1 Q79H mutant
Pro1 Q79H Rv	CTGCGATGGCGTGAACTTCTGCT	
Pro1 Q79K Fw	AGCAGAAGTTAAGGCCATCGCAG	For construction of Pro1 Q79K mutant
Pro1 Q79K Rv	CTGCGATGGCCTTAACTTCTGCT	
Pro1 Q79N Fw	AGCAGAAGTTAACGCCATCGCAG	For construction of Pro1 Q79N mutant
Pro1 Q79N Rv	CTGCGATGGCGTTAACTTCTGCT	
Pro1 Q79R Fw	AGCAGAAGTTCGGGCCATCGCAG	For construction of Pro1 Q79R mutant
Pro1 Q79R Rv	CTGCGATGGCCCGAACTTCTGCT	
Pro1 Q79W Fw	AGCAGAAGTTTGGGCCATCGCAG	For construction of Pro1 Q79W mutant
Pro1 Q79W Rv	CTGCGATGGCCCAAACTTCTGCT	
Pro1 I150T Fw	ACACTATCTGTTAGAGAAACCAAATTTGGT	For construction of Pro1 I150T mutant
Pro1 I150T Rv	GTTAGAGAAACCAAATTTGGTGACAATGAC	
Pro1 D143A Fw	GTGAATGAAAACGCCACACTATC	For construction of Pro1 D143A mutant
Pro1 D143A Rv	GATAGTGTGGCGTTTTCATTCAC	

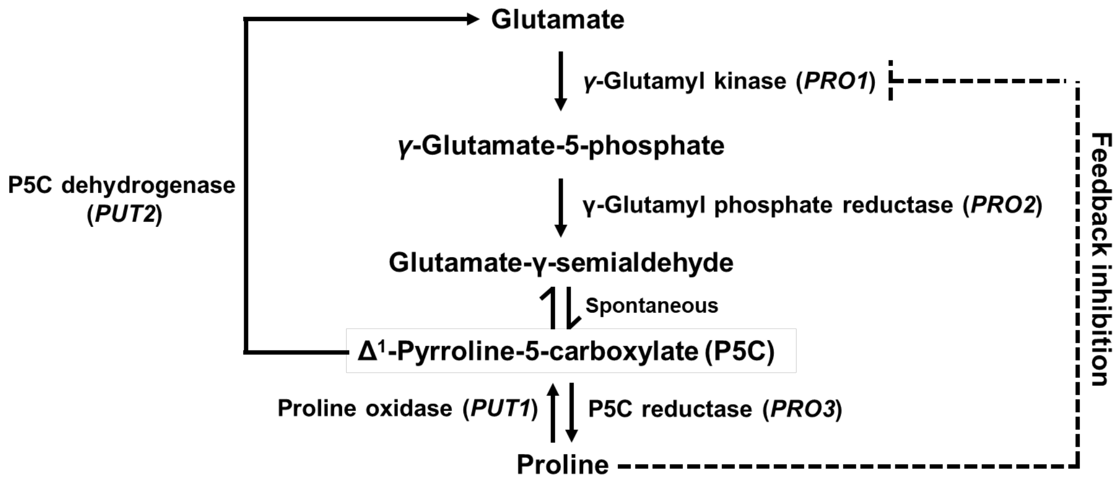

Figure S1. Biosynthesis and degradation of proline. In *S. cerevisiae*, proline is synthesized from glutamate by three cytoplasmic enzymes: the γ-glutamyl kinase Pro1, the γ-glutamyl phosphate reductase Pro2, and the Δ^1-pyrroline-5-carboxylate reductase Pro3. On the other hand, proline is oxidized to P5C by the mitochondrial proline oxidase Put1. P5C is then converted into glutamate by the P5C dehydrogenase Put2. The genes which encode enzymes are indicated in parentheses.

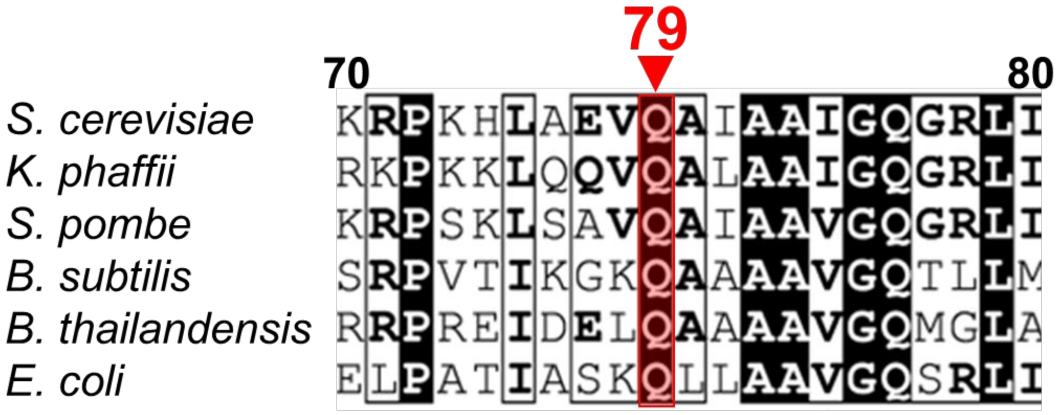

Figure S2. Sequence alignment around Gln79 in Pro1 among various microorganisms. The amino acid sequence of the *Saccharomyces cerevisiae* (*S. cerevisiae*) Pro1 was compared to that of the Prol homologues from *Komagataella phaffii* (*K. phaffii*), *Schizosaccharomyces pombe* (*S. pombe*), *Bacillus subtilis* (*B. subtilis*), *Burkholderia thailandensis* (*B. thailandensis*), and *Escherichia coli* (*E.coli*). Numbering of residues is in the *S. cerevisiae* Pro1 and conserved residues were highlighted in black boxes. Gln79 is shown in red.

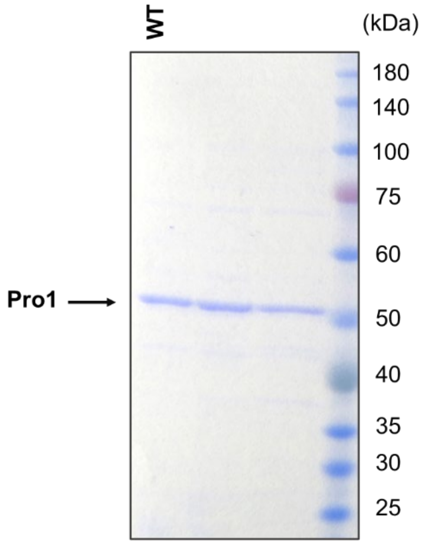

Figure S3. Purity of the recombinant Pro1 (WT, Q79H, and I150T) proteins. The purified Pro1 variants were subjected to SDS-polyacrylamide gel electrophoresis (10%) and were stained with Coomassie Brilliant Blue. Marker: molecular mass standard.

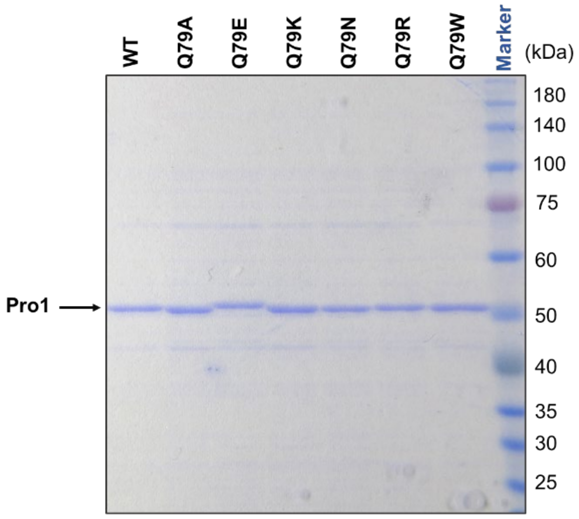

Figure S4. Purity of the recombinant Pro1 (WT, Q79A, Q79E, Q79K, Q79N, Q79R, and Q79W) proteins. The purified Pro1 variants were subjected to SDS-polyacrylamide gel electrophoresis (10%) and were stained with Coomassie Brilliant Blue. Marker: molecular mass standard.

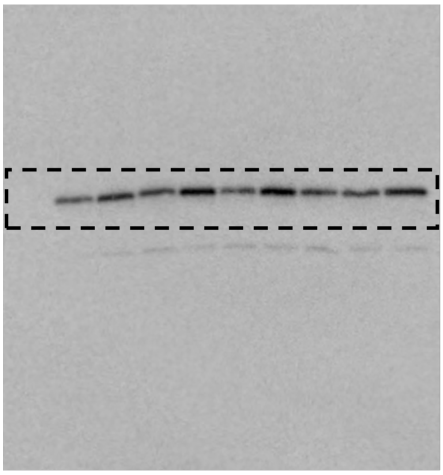

α-HA

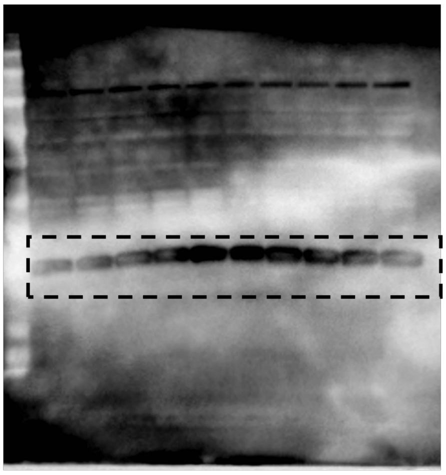

α-GAPDH

Figure S5. Uncropped images for Western blot gels of Figure 5 (a). Broken boxes mark the borders of the final cropped images.

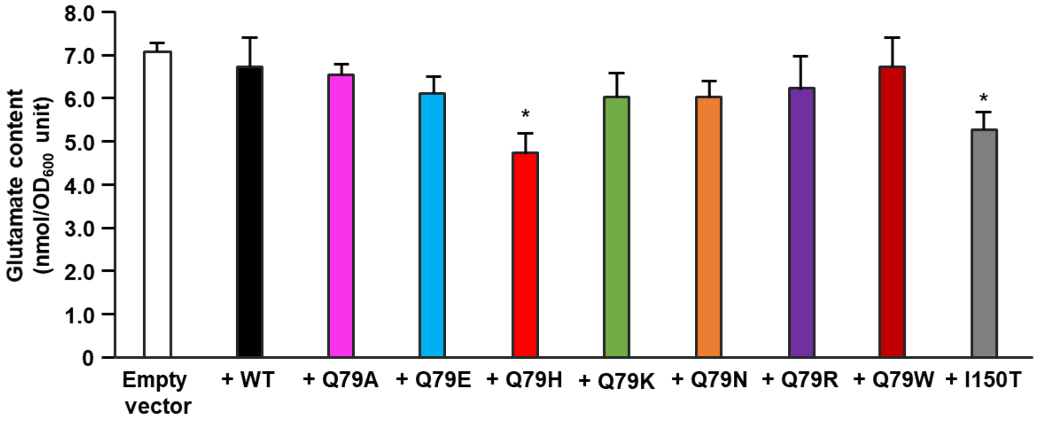

Figure S6. Glutamate contents in yeast cells expressing the Gln79 variants of Pro1. The Wild-type (+ WT), Q79A (+ Q79A), Q79E (+ Q79E), Q79H (+ Q79H), Q79K (+ Q79K), Q79N (+ Q79N), Q79R (+ Q79R), or Q79W (+ Q79W) variant Pro1 was expressed in a laboratory strain BY4741. Each strain was grown on SD+Am medium and intracellular glutamate contents were determined. Empty vector indicates a negative control.

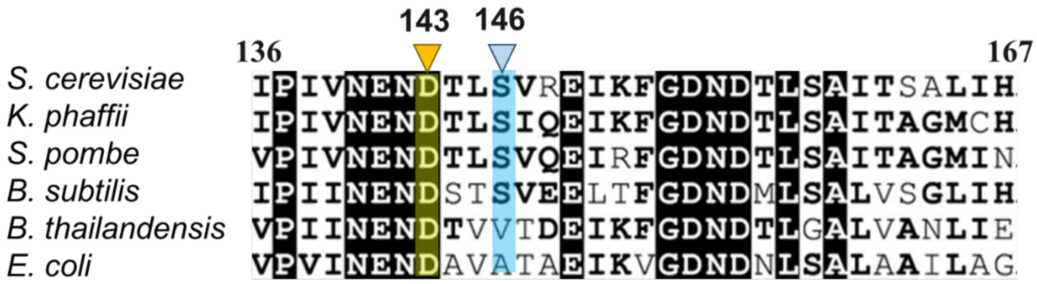

Figure S7. Sequence alignment around Asp143 and Ser146 in Pro1 among various microorganisms. The amino acid sequence of *Saccharomyces cerevisiae* (*S. cerevisiae*) Pro1 was compared to that of the Prol homologues from *Komagataella phaffii* (*K. phaffii*), *Schizosaccharomyces pombe* (*S. pombe*), *Bacillus subtilis* (*B. subtilis*), *Burkholderia thailandensis* (*B. thailandensis*), and *Escherichia coli* (*E.coli*). Numbering of residues is in the *S. cerevisiae* Pro1 and conserved residues were highlighted in black boxes. Asp143 and Ser146 are shown in yellow and light blue, respectively.

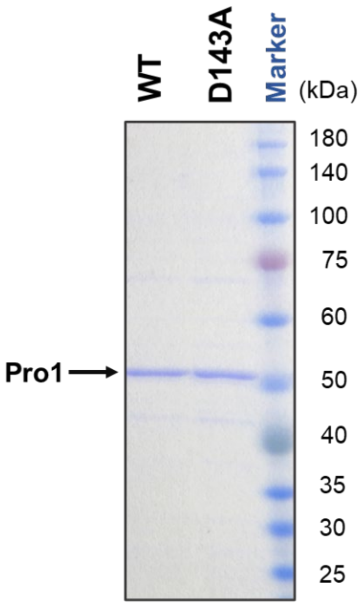

Figure S8. Purity of the recombinant Pro1 (WT and D143A) proteins. The purified Pro1 variants were subjected to SDS-polyacrylamide gel electrophoresis (10%) and were stained with Coomassie Brilliant Blue. Marker: molecular mass standard.
